# Epstein-Barr Virus Infection-Associated Facial Swelling: A Case Report

**DOI:** 10.7759/cureus.57635

**Published:** 2024-04-04

**Authors:** Yue Zhang, Jinlong Chen, Liangrui Chen, Wei Tang

**Affiliations:** 1 Department of Oral and Maxillofacial Surgery, State Key Laboratory of Oral Diseases & National Clinical Research Center for Oral Diseases, West China Hospital of Stomatology, Sichuan University, Chengdu, CHN

**Keywords:** clinical diagnosis, genomic testing, maxillofacial infection, epstein-barr virus infection, facial edema

## Abstract

Maxillofacial soft tissue swelling is a common clinical symptom with various etiologies. While odontogenic space infection is the most common cause, it is crucial not to overlook maxillofacial swellings caused by specific pathogenic infections and other local factors. This paper reports the case of an adult patient with right-sided swelling of his face, persistent oral mucosal ulcers, and recurrent hyperthermia for 30 days. He had received various antibiotics for the initial diagnosis of "right buccal space infection," but the antibiotics did not have any effect on his symptoms. None of the blood tests, histological examinations, bone marrow biopsies, and immune-related tests produced diagnostic findings. A diagnosis of Epstein-Barr virus (EBV) infection was finally confirmed by biopsy tissue genomics sequencing and quantitative analysis of EBV nucleic acid. In this report, we describe the diagnosis and treatment process for this patient and suggest that facial swelling could be an important clinical symptom of EBV infection.

## Introduction

Human gammaherpesvirus type 4, also known as Epstein-Barr virus (EBV), is an enveloped double-stranded deoxyribonucleic acid (DNA) virus [1]. EBV is arguably the most ubiquitous of human viruses, and it infects about 90% of adults worldwide. It is most commonly transmitted by contact with respiratory secretions. Primary EBV infection is the leading cause of infectious mononucleosis [2]. The typical triad symptoms of EBV infection include fever, pharyngitis, and lymphadenopathy. However, in patients over 35 years of age, the clinical manifestations are atypical and may present as infection, unexplained fever, or some rare complications, including airway obstruction due to oropharyngeal inflammation, meningoencephalitis, treptococcal pharyngitis, hemolytic anemia, and other diseases [3]. It has been suggested that EBV infection is implicated in the pathogenesis of Hodgkin's lymphoma, nasopharyngeal carcinoma, and a myriad of malignancies [2]. EBV infection usually leads to bilateral temporal and periorbital swelling [4], but no clinical cases of unilateral buccal soft tissue swelling caused by EBV infection have been reported. This paper reports a case of right buccal swelling caused by EBV in an adult and discusses it in combination with the relevant literature.

## Case presentation

A 55-year-old male presented at our outpatient clinic with pain in the right upper posterior teeth region accompanied by swelling and tenderness of the right buccal region for more than 10 days, with his symptoms progressively worsening. The patient had a two-year history of myelofibrosis and had taken ruxolitinib hydrochloride tablets, hydroxyurea tablets, and aspirin for two years, which had been discontinued because of his maxillofacial swelling. He denied a history of trauma or allergies. Physical examination revealed limited mouth opening without dyspnea or dysphagia and obvious asymmetric swelling of the patient's right buccal region extending up to the zygomatic arch and down to the lower margin of the mandible, which was characterized by surface redness, firmness on palpation, and elevated skin temperature. Multiple lymph nodes in the right neck were enlarged with poorly defined borders and firm quality, and the right buccal mucosa was markedly red and swollen intraorally. The upper-right third molar had grade III tooth mobility. Considering the diagnosis of a "right maxillofacial space infection," he was prescribed amoxicillin for oral antibacterial treatment.

**Figure 1:**
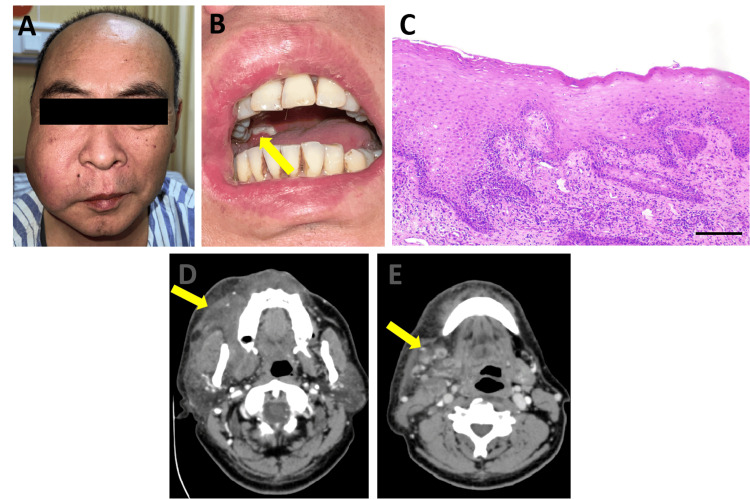
Physical examination and auxiliary tests before admission (A) Facial appearance of the patient on his first day of hospitalization. (B) Limited mouth opening and nonhealing of the socket of the tooth that was extracted. (C) HE staining of the buccal mucosa biopsy tissue suggesting inflammatory lesions (magnification, 10x). Enhanced CT section images at the buccal level (D) and neck level (E). HE: Hematoxylin-eosin; CT: Computed tomography

Five days later, swelling of the right buccal region had not been ameliorated. The upper-right third molar was then extracted for drainage, and an incisional biopsy was taken at the buccal mucosa site around the tooth extraction socket. Seven days after the upper-right third molar extraction, all the symptoms persisted (Figure 1A). The tooth extraction socket did not heal at all. An unpainful ulcer of the same shape as the biopsy site was seen on the right buccal mucosa connected to the extraction socket, which was covered with yellowish-white pseudomembrane, and the surrounding mucosa was hyperemic, slightly red and swollen (Figure 1B). Biopsy revealed slight hyperplasia of the right buccal mucosal epithelium, edema of epithelial surface cells, ballooning degeneration, and massive acute and chronic inflammatory cell infiltration of the lamina propria, involving the muscular layer (Figure 1C). Enhanced computed tomography (CT) also revealed linear heterogeneous enhancement in the concave area of the right buccal region after enhancement, thickening and swelling of soft tissue in the right buccal space and right parotid masseter area, widening of the space, and a low-density liquefaction interval in the right mandibular area, suggesting right buccal tumor changes or abscess formation in the right maxillofacial area (Figure 1D). The patient had multiple enlarged lymph nodes in the right neck Ib and II areas, with heterogeneous density and enhancement, which suggested local tumor metastasis (Figure 1E). Based on the patient's medical history and pathological and imaging results, the following diagnosis was considered: "right buccal space infection," and the patient was admitted.

**Figure 2:**
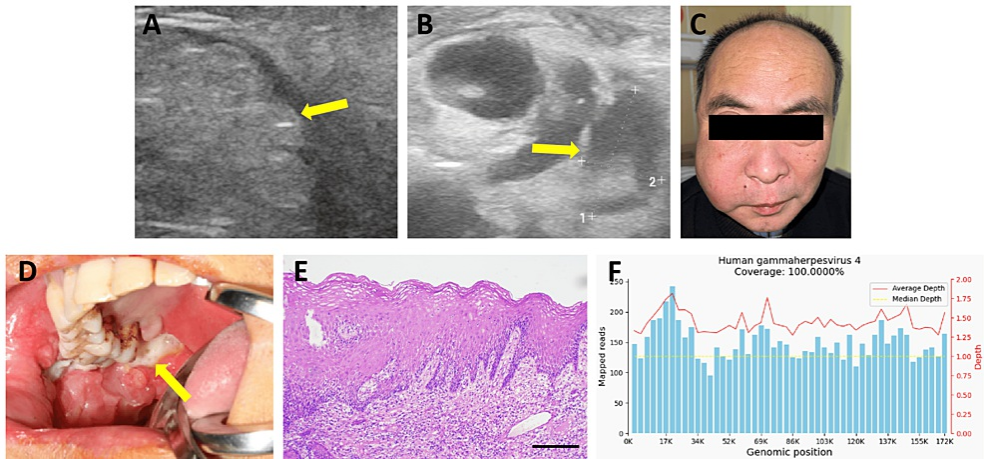
Physical examination and auxiliary tests after admission Color Doppler ultrasound of the right buccal region (A) and the right neck (B). (C) The facial appearance of the patient after hormone shock therapy. (D) Intraoral image before the second biopsy. (E) HE staining of the buccal mucosa second biopsy tissue suggests the presence of inflammatory lesions (magnification, 10x). (F) EBV was detected by mNGS. HE: Hematoxylin-eosin; EBV: Epstein-Barr virus; mNGS: metagenomics next-generation sequencing

Hematological examination revealed a normal white blood cell count (WBC), the monocyte (MONO%) was 10.70%, the lymphocyte (LYM%) was 16.60%, and his hypersensitive C-reactive protein (hs-CRP) level was 16.92 mg/L, suggesting the presence of inflammation. The patient was empirically prescribed piperacillin sodium, tazobactam sodium, and vancomycin for anti-infective therapy. At the same time, faciocervical color Doppler ultrasound revealed thickening of the subcutaneous tissue of the right faciocervical region and parotid gland and masseter muscle, with fine and enhanced echoes and slit-like anechogenicity, suggesting the possibility of right maxillofacial space infection (Figure 2A). Several lymph node echoes were found in area II of the right neck, approximately 16.5 mm × 15.8 mm, with poor boundaries, irregular shapes, and an unclear corticomedullary demarcation (Figure 2B). The results showed that the right facial lesions were atypical and the risk of needle biopsy of the right cervical lymph nodes was high, so needle biopsy was not planned for the time being. The results of the above examinations were not definitive; the possibility of infection, lymphoma, or systemic disease could not be excluded; and targeted examinations were planned.

In the following days, the patient had a persistent fever of up to 39.9°C, which did not respond significantly to physical cooling and paracetamol suspension. The swelling of the right maxillofacial region was not significant. Also, antibiotic treatment was ineffective; therefore, the antibiotic treatment was stopped. The bacterial culture results of the secretions from the biopsy site were negative. Then, a puncture was performed at the right buccal region, and no pus was found. Based on the results of antibiotic treatment, bacterial culture, and puncture at the right buccal region, the possibility of maxillofacial space infection was excluded.

Bone marrow aspiration cytology revealed that the bone marrow was actively proliferating, with 50% of the cells being granulocytes and 43.5% of the cells being erythroid, and there were no increases in progenitor cells, which excluded the possibility of lymphoma.

The patient had recurrent high fever without obvious symptoms. Based on the other tests, the possibility of connective tissue disease was considered, and the patient was given prednisone tablets for three days of hormone shock therapy. The swelling in the right maxillofacial region had slightly improved after treatment (Figure 2C). Immune-related tests were also performed, and the results showed that the total immunoglobulin E (IgE) concentration was 335.00 IU/mL, the immunoglobulin G4 subtype was 2.060 g/L, and no significant abnormalities were found, which excluded the possibility of rheumatic immune diseases such as a connective tissue disease.

To summarize the case thus far, infectious diseases caused by special pathogens were still considered, and the diagnosis was revised to a "right facial infection (mass with infection?)." Pathological examination was planned after a second biopsy of the lesion site was taken. Under general anesthesia, an excisional biopsy of the right buccal and soft palate lesions was performed, during which the ulcers on the right cheek and right maxillary soft palate did not change significantly compared with their characteristics on admission (Figure 2D). Pathology revealed severely inflamed mucosal tissue in the right buccal region, in which large areas of necrosis were observed, and the inflammation had spread to the muscle layer. Ulceration was observed in the right soft palate mucosa, and many inflammatory cells were densely infiltrated in the lamina propria, which suggested inflammatory lesions (Figure 2E) and excluded the possibility of maxillofacial tumors.

At the same time, the biopsy samples were sent for genomic sequencing. The pathogen capture metagenomics next-generation sequencing (mNGS) revealed that common microecological flora, human gammaherpesvirus 4 (number: 13,434), and thin-loop virus (number: 7) were detected in the submitted tissues (Figure 2F). EBV infection was considered, and real-time fluorescence detection of EBV nucleic acid was performed, with a result of 4.21 × 10^3^ copies/mL, which confirmed the diagnosis of right maxillofacial swelling caused by EBV infection. It was suggested that the patient be treated in the infectious disease department for chronic EBV infection and given ganciclovir 5 mg/kg intravenously. Two weeks after antiviral therapy, the patient's general condition improved, with a further reduction of swelling in the right maxillofacial region, and he has remained under follow-up.

**Figure 3:**
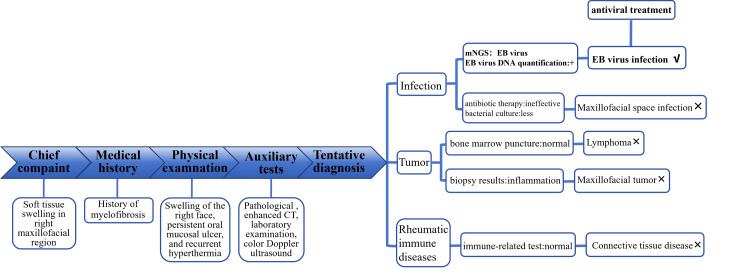
The clinical diagnosis process for the patient EB virus: Epstein-Barr virus; mNGS: metagenomics next-generation sequencing; CT: Computed tomography; DNA: Deoxyribonucleic acid

The diagnostic process of the patient is described in Figure 3.

## Discussion

Maxillofacial soft tissue swelling is very common in clinical practice, and its etiology mainly includes local factors and systemic factors. Diseases such as congestive heart failure, renal failure, or liver cirrhosis usually lead to fluid retention and general facial swelling [5,6]. Maxillofacial swelling caused by local factors mostly occurs unilaterally, such as in the following scenarios: (i) Infections: bacterial, viral, or fungal infections of soft tissues, including salivary glands, can lead to maxillofacial swelling and tenderness, among which odontogenic space infections are the most common [7]. (ii) Tumors: benign or malignant tumors originating from maxillofacial soft tissue cause progressive swelling of the maxillofacial region. (iii) Trauma: trauma in the maxillofacial region can cause edema or hematoma of maxillofacial tissues [8].

A correct diagnosis for the cause of soft tissue swelling in the maxillofacial region requires (i) a thorough medical history, including the patient’s recent past history and personal history; (ii) a general physical assessment, assessing the patient for signs and symptoms of an underlying systemic disease such as fatigue and fever, while considering performing thorough examinations of other body systems to identify any possible systemic involvement; and (iii) a comprehensive physical examination, paying attention to the swelling location, scope, mobility, tenderness, and the presence of associated symptoms such as numbness or lymphadenopathy. We evaluated whether there were signs of infection, abscess, or other abnormalities in the teeth, gums, or surrounding tissues.

The diagnosis range can be narrowed based on the history and physical examination, but maxillofacial soft tissue swelling caused by different etiologies often has similar accompanying symptoms, causing the rational use of various auxiliary examinations to further confirm the diagnosis to be a challenge in clinical work. (i) Targeted laboratory tests, appropriate blood or other body fluid tests, and bacterial culture can be performed to evaluate autoimmune diseases or tissue and organ dysfunction or to clarify whether there are inflammatory indicators and signs of infection, including specific tests based on suspected systemic diseases. (ii) Head and neck imaging examination, using cone beam computed tomography (CBCT) and enhanced CT scanning can be used to visualize the swelling area and surrounding lymph node structure. If low-density liquefied areas are observed on these imaging tests, the possibility of local infection is greater, and maxillofacial tumorigenesis is more likely to occur if soft tissue or lymph node enhancement occurs. If the diagnosis remains unclear, color Doppler ultrasound can be performed in the swollen areas and lymph nodes, and the differential diagnosis can be made according to regional blood flow and soft tissue echo guidance. (iii) Empirical use of drugs: a maxillofacial space infection can also be confirmed when the swelling responds to the empirical use of antibiotics in the early stage. If antibiotic treatment is ineffective, other viruses or fungi that can cause infection or maxillofacial tumors should be considered. (iv) Fine needle aspiration or biopsy/removal of biopsy tissue: when an accurate diagnosis is not obtained with the above examinations or if there is a suspected infection caused by tumors or special pathogens, the use of pathological examination or immunohistochemical staining, which is usually the gold standard for diagnosis, should be considered to clarify the cell types of the lesion area. (5) Genomics testing, which also plays an increasingly important role in the detection of specific pathogens, includes mNGS, which is a method for identifying and analyzing the pathogens present in biological samples (such as blood, saliva, or tissue) [9]. mNGS can capture and sequence the genetic material of all microorganisms (including bacteria, viruses, fungi, and parasites) from a sample, is a comprehensive and sensitive detection method, and can simultaneously detect a variety of pathogens, including EBV, even if the virus concentration is low or if the virus is mixed with other pathogens [10].

**Figure 4:**
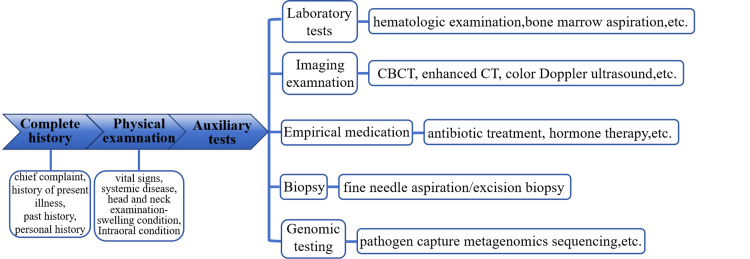
General diagnostic procedure for maxillofacial soft tissue swelling CBCT: Cone beam computed tomography; CT: Computed tomography

The general diagnostic process for maxillofacial soft tissue swelling is shown in Figure 4.

In this case, the patient complained of right maxillofacial swelling rather than extensive maxillofacial swelling. After comprehensive consideration of the patient's personal history and past history, the diagnosis of maxillofacial swelling caused by allergies and a history of myelofibrosis were excluded. According to the results of physical examination, pathology, enhanced CT, and color Doppler ultrasound, the proposed diagnosis was maxillofacial space infection. The patient failed to respond to antibiotic treatment, and this diagnosis could not be completely supported by the patient’s hemogram and bacterial culture results. Maxillofacial swelling can be caused by infections caused by special pathogens, lymphoma, a connective tissue disease, or another rheumatic immune system disease. Therefore, further examinations were needed in this case to clarify the etiology. Bone marrow aspiration cytology and immune-related tests were performed to exclude the above-suspected etiology. A second biopsy of the right buccal and soft palate mucosa was performed along with pathological examination and metagenomic testing, which revealed EBV infection, and quantification of the amount of EBV nucleic acid in the samples finally confirmed the diagnosis of maxillofacial soft tissue swelling caused by EBV.

EBV is one of the most common viruses in humans. In most cases, EBV infections are mostly self-limiting and have no complications. When symptoms do occur, a variety of symptoms can be observed. The most frequently reported complications that involve the maxillofacial region include Hodgkin's lymphoma and nasopharyngeal carcinoma [11-13]. Typical features of EBV infection include lymphadenopathy, fever, fatigue, and pharyngitis. Cervical lymph node enlargement can lead to extensive maxillofacial swelling, but it is not common and is often accompanied by systemic symptoms such as recurrent fever [14]. The diagnosis of EBV infection is usually based on clinical symptoms, physical examination results, and laboratory tests, and quantitative analysis of nucleic acid is widely used in the diagnosis of EBV [15]. Hematological examinations can also assist in the diagnosis of the disease by the detection of specific antibodies produced by the immune system against the virus, and these antibodies include heterophile antibodies (such as single spot tests) and EBV-specific antibodies. There is no specific treatment for EBV infection, and conventional antiviral therapy and symptomatic and supportive treatment are usually administered [16].

## Conclusions

Unilateral maxillofacial soft tissue swelling caused by EBV is very rare in clinical practice and is often misdiagnosed as a maxillofacial space infection, tumor, or other disease. When maxillofacial soft tissue swelling is found, the possibility of maxillofacial infection caused by special pathogens such as EBV cannot be ignored. After combining the patient's systemic symptoms and the results of various recommended examinations, a clear diagnosis should be made and targeted treatment should be given to the patients.
